# Structure and mechanism of copper–carbonic anhydrase II: a nitrite reductase

**DOI:** 10.1107/S2052252520000986

**Published:** 2020-02-21

**Authors:** Jacob T. Andring, Chae Un Kim, Robert McKenna

**Affiliations:** a Department of Biochemistry and Molecular Biology, College of Medicine, University of Florida, Gainesville, FL 32610 USA; b Department of Physics, Ulsan National Institute of Science and Technology (UNIST), Ulsan 44919, Republic of Korea

**Keywords:** catalytic metal ions, copper–carbonic anhydrase II, apo-carbonic anhydrase II, nitrite reductases, nitric oxide, X-ray crystallography

## Abstract

Nitric oxide (NO) promotes vasodilation through the activation of guanylate cyclase, resulting in the relaxation of the smooth muscle vasculature and a subsequent decrease in blood pressure. Therefore, its regulation is of interest for the treatment and prevention of heart disease. An example is pulmonary hypertension which is treated by targeting this NO/vasodilation pathway. In bacteria, plants and fungi, nitrite (NO_2_^−^) is utilized as a source of NO through enzymes known as nitrite reductases. These enzymes reduce NO_2_^−^ to NO through a catalytic metal ion, often copper. Recently, several studies have shown nitrite reductase activity of mammalian carbonic anhydrase II (CAII), yet the molecular basis for this activity is unknown. Here we report the crystal structure of copper-bound human CAII (Cu–CAII) in complex with NO_2_^−^ at 1.2 Å resolution. The structure exhibits Type 1 (T-1) and 2 (T-2) copper centers, analogous to bacterial nitrite reductases, both required for catalysis. The copper-substituted CAII active site is penta-coordinated with a ‘side-on’ bound NO_2_^−^, resembling a T-2 center. At the N terminus, several residues that are normally disordered form a porphyrin ring-like configuration surrounding a second copper, acting as a T-1 center. A structural comparison with both apo- (without metal) and zinc-bound CAII (Zn–CAII) provides a mechanistic picture of how, in the presence of copper, CAII, with minimal conformational changes, can function as a nitrite reductase.

## Abstract 2

This work utilizes X-ray crystallography to provide mechanistic insights into how carbonic anhydrase II can act as a nitrite reductase in the presence of copper.

## 1. Introduction   

In mammals (including humans), it has been well established that zinc–carbonic anhydrase (Zn–CA) catalyzes the reversible hydration/dehydration of carbon dioxide (CO_2_)/bicarbonate (HCO_3_^−^) (Meldrum & Roughton, 1933 ▸; Stadie & O’Brien, 1933 ▸). There are 12 enzymatically active CA isoforms in humans, with CAI and II abundant in most cells, especially in red blood cells (RBCs), and as such they are directly involved in gas exchange, ion transport, and extra- and intracellular pH regulation (Frost, 2014 ▸). A single Zn–CAI or II protein is capable of converting ∼0.2 and 1.1 × 10^6^ CO_2_ to HCO_3_^−^ per second, respectively (Supuran, 2008 ▸; Steiner *et al.*, 1975 ▸). Hence, with a concentration of Zn–CAI and II of 4.2 × 10^6^ and 4.8 × 10^5^ molecules per RBC, there are excessive amounts of CA to regulate the 5 × 10^20^ CO_2_ generated in an adult human breath (Moini *et al.*, 2002 ▸). This excess of CA in the blood leads to the question do carbonic anhydrases have other regulatory roles? Many reports have shown CAII is a promiscuous enzyme, capable of binding multiple substrates and performing a variety of reactions besides its carbonic anhydrase activity. These include binding other gaseous molecules such as nitrate, nitrite and molecular oxygen; esterase activity with many ester-containing compounds; and hydration reactions such as hydrating cyanamide to urea (Briganti *et al.*, 1999 ▸; Nielsen & Fago, 2015 ▸; Mangani & Håkansson, 1992 ▸; Piazzetta *et al.*, 2017 ▸). While these activities are important and show CAII’s robust role, recent reports have shown that CAII can also reduce nitrite (NO_2_^−^ **)** to nitric oxide (NO), and thus, may play a role in vasodilation and regulation of blood pressure (Andring *et al.*, 2018 ▸; Aamand *et al.*, 2009 ▸; Hanff *et al.*, 2018 ▸).

**Figure 1:**
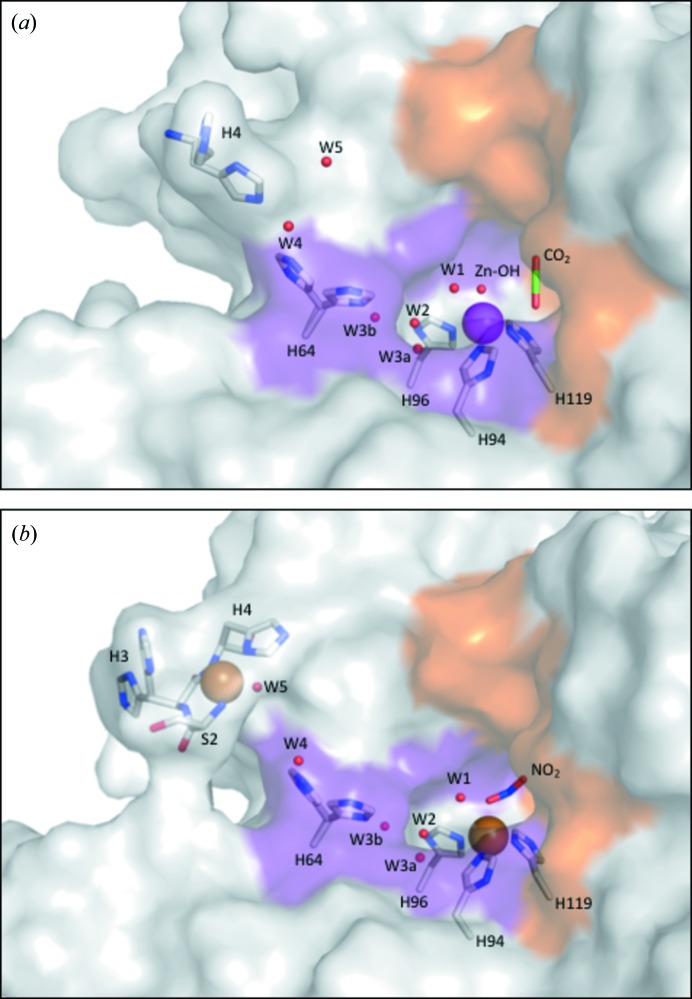
Structure of Zn– and Cu–CAII: active site and water network. (*a*) Zn–CAII. The zinc metal is stabilized by three histidines (His94, His96 and His119). His64 is depicted in its dual ‘in’ and ‘out’ conformations. The N terminus (residues 1–4) is disordered. The substrate CO_2_ is shown bound adjacent to the zinc, stabilized by the hydro­phobic pocket (Domsic *et al.*, 2008 ▸). (*b*) Cu–CAII. The copper metal (T-2 site) is stabilized by the same three histidines as the zinc. Also, His64 was observed in dual conformations. The NO_2_^−^ bound with an oxygen and a nitro­gen interacting with the copper. The water network resembles that observed in Zn–CAII, with the exception of the extended water network (W4 and W5), creating a hydrogen-bonding network spanning from the N terminus to the active site. The N terminus is ordered, forming a pseudo-porphyrin ring around a second copper (T-2 site). The catalytic metals are depicted as spheres: zinc (magenta) and copper (brown). The hydro­phobic residues (Ile91, Val121, Phe131, Val135, Leu141, Val143, Leu198, Pro202, Leu204, Val207 and Trp209) are highlighted as orange and the hydro­philic residues (Asn62, His64, Asn67, Gln92, Thr199 and Thr200) as violet. The active-site solvent network (W1, W2, W3a and W3b) is depicted as red spheres and the extended water network (W4 and W5) is shown in the Cu–CAII substituted structure.

CAII is a 30 kDa protein, with a solvent-exposed active site. In Zn–CAII, the zinc is tetrahedrally coordinated by three histidines (His94, His96, and His119) and a solvent molecule. The active site is divided into a distinct hydro­phobic and hydro­philic side. The hydro­phobic side (residues Ile91, Val121, Phe131, Val135, Leu141, Val143, Leu198, Pro202, Leu204, Val207 and Trp209) stabilizes the CO_2_ substrate, while the hydro­philic side (Asp62, His64, Asp67, Gln92, Thr199 and Thr200) orders and regulates the solvent [W1, W2, W3a, W3b and WD (deep water)] required for rapid catalytic turnover (Frost, 2014 ▸). Of special importance is His64, that modulates between an ‘in’ and ‘out’ conformation (referring to its direction relative to the active site), and is known to be important in proton transfer [Fig. 1 ▸(*a*)] (Fisher *et al.*, 2011 ▸). Of note, all deposited structures of Zn–CAII to date have disordered N termini (residues 1–4). The role of Zn–CAII in the hydration/dehydration of CO_2_/HCO_3_^−^ has been extensively studied. The reaction is a two-step ping-pong mechanism. In the hydration direction, the first step is the nucleophilic attack of CO_2_ by a zinc-bound hydroxyl that results in the formation of HCO_3_^−^, which is displaced by a water molecule (Domsic *et al.*, 2008 ▸). The second step of the reaction is the transfer of a proton from the zinc-bound water to the bulk solvent via the well defined solvent network and His64 (Silverman & Lindskog, 1988 ▸). The regeneration of the zinc-bound hydroxyl permits the catalytic reaction cycle, the *k*_cat_/*K*_m_ of the reaction is 120 *M* ^−1^ µs^−1^, which means Zn–CAII has evolved to near catalytic perfection for the hydration/dehydration of CO_2_/HCO_3_^−^, as it is diffusion-rate limited (Maupin *et al.*, 2009 ▸).

In humans, the most common source of NO is its synthesis by endothelial nitro­gen oxide synthase (eNOS), which catalyzes the oxidation of arginine to produce NO and citrulline (Feng, 2012 ▸). While this enzyme is responsible for NO production under normoxia, under hypoxic conditions the enzyme is acatalytic (Lundberg *et al.*, 2008 ▸). Thus, other less understood pathway(s) have been suggested to function in the place of eNOS during times of low oxygen to produce NO through a nitrite reduction pathway. Nitrite represents an untapped source of NO in the blood with little understanding of how it is reduced. Although, previous studies have suggested hemoglobin or a CA as likely candidates as the nitrite reductase (Lundberg *et al.*, 2008 ▸; Sparacino-Watkins *et al.*, 2012 ▸).

Bacterial copper nitrite reductases utilize two separate and distinct copper-binding sites to catalyze the reduction of nitrite. The first copper site, known as the Type I (T-1) site and coordinated by a cysteine, a me­thio­nine and two histidines, functions to transfer electrons to the second copper site termed the Type II (T-2) site (see Fig. S1 in the supporting information) (Li *et al.*, 2015 ▸). The T-2 site, coordinated by three histidines and a solvent molecule, is where the nitrite reduction reaction occurs (Li *et al.*, 2015 ▸). It is interesting to note that previous studies have commented on the striking similarity between the Zn–CAII active site and bacterial nitrite reductase T-2 sites, suggesting that CAII may be involved in mammalian nitrite reduction (Strange *et al.*, 1995 ▸). In addition, recent studies have shown that bovine CAII can reduce NO_2_^−^ to NO (Aamand *et al.*, 2009 ▸). However, when dialyzed with ethylenediaminetetraacetic acid (EDTA), the enzyme retained its carbonic anhydrase activity yet lost its nitrite reductase activity (Hanff *et al.*, 2018 ▸). While zinc is a divalent cation, it has a full *d* orbital when coordinated in CAII, and thus, is unable to perform redox reactions. The two observations, taken together, suggest that there may be a factor in blood that activates the nitrite reductase activity of CAII. In blood, there is a relatively high concentration of copper (∼15 µ*M*), which can replace the zinc in the CAII active site, as previous research has shown that CAII preferentially binds copper with 50-fold specificity over zinc (Schultze *et al.*, 2014 ▸; Håkansson *et al.*, 1994 ▸; Hunt *et al.*, 1999 ▸). Hence, based on the knowledge of copper-containing bacterial nitrite reductases, we hypothesized that copper was the additional cofactor in blood responsible for the nitrite reductase activity of CAII previously reported. Therefore, the addition of copper to apo-CAII (without metal) could be the mechanism to convert CAII to a nitrite reductase. In this study, we compared the crystal structures of Cu– with both apo- and Zn–CAII (see Methods[Sec sec4]) to obtain a mechanistic picture of how, in the presence of copper, CAII can, with minimal conformational changes, be converted into a nitrite reductase.

## 2. Results   

Mammalian CAs selectively use zinc as their catalytic metal ion, using it as a Lewis acid to increase the nucleophilic character of the zinc-bound hydroxyl. The CA active site, as described above, has the same characteristics as a T-2 copper-binding site in bacterial nitrite reductases: three coordinating histidines, polar residues for charged transition-state stabilization and metal-bound solvent molecules. Our crystal structure confirmed this, with the Cu–CAII T-2 site having the same general conformation as the zinc active site. The copper atom is penta-coordinated via the three histidine residues (His94, His96 and His119), and a nitrite molecule, bound in a ‘side-on’ conformation, coordinated via an oxygen and a nitro­gen (Fig. 1 ▸). The copper-substituted active site forms a T-2 site perfectly, as described in bacterial copper nitrite reductases.

**Figure 2:**
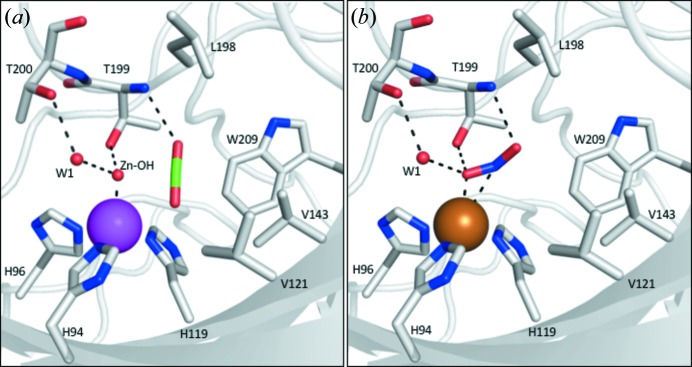
Active site of Zn– and Cu–CAII (T-2 site) with bound substrate, CO_2_ and NO_2_^−^, respectively. (*a*) CO_2_ binding site in Zn–CAII active site (adapted from PDB entries 3ks3, Avvaru *et al.*, 2010 ▸; 5yui, Kim *et al.*, 2016 ▸). CO_2_ binds adjacent to the zinc, ∼2.8 Å from the catalytic zinc-bound solvent. The CO_2_ is stabilized via interactions with residues Val121, Val143, Leu198 and Trp209. Thr199 also forms a hydrogen bond with CO_2_ via its nitro­gen. (*b*) NO_2_^−^ binding site in Cu–CAII active site. NO_2_^−^ binds directly to the copper, displacing the copper-bound solvent. It binds in a ‘side-on’ conformation via an oxygen and a nitro­gen, 2.1 and 2.8 Å from the copper, respectively. However, solvent W1 retains its position and forms hydrogen bonds with an oxygen of NO_2_^−^. Thr199 forms two hydrogen bonds with the bound NO_2_^−^ while Leu198 also forms stabilizing interactions. The catalytic metals are depicted as spheres: zinc (magenta) and copper (brown). The active-site solvent molecules are depicted as red spheres.

As mentioned previously, an ordered water network exists within Zn–CAII, responsible for rapid proton transfer [Fig. 1 ▸(*a*)]. In the active site, the Cu–CAII has a slightly altered water network compared with the Zn–CAII structure [Fig. 1 ▸(*b*)] in that the zinc-bound solvent is replaced with a bound NO_2_^−^ molecule in a ‘side-on’ configuration (Fig. 2 ▸). This was unexpected, as no nitrite or nitro­gen source was added to the crystallization conditions (1.6 *M* sodium citrate and 50 m*M* Tris at a pH of 7.8). However, previous structural studies of bacterial copper nitrite reductases have revealed endogenous ligands bound to the T-2 site (Antonyuk *et al.*, 2005 ▸; Fukuda *et al.*, 2015 ▸). Both NO_2_^−^ and NO have been shown to be bound in the *Achromobacter cyclo­clastes* T-2 copper site without being added to the crystal (Fig. S1). While the origin of these ligands is unknown, others have hypothesized synchrotron radiation as a possible source of high-energy ions leading to the formation of these molecules (Fukuda *et al.*, 2015 ▸). One of the NO_2_^−^ oxygens occupies the position of the Zn–CAII deep water, which is important for solvent replenishment (Kim *et al.*, 2016 ▸, 2018 ▸). Comparison of the Zn–CAII:CO_2_ complex (PDB entry 5yui, Kim *et al.*, 2016 ▸) with the Cu–CAII:NO_2_^−^ complex shows significant differences. While the NO_2_^−^ binds directly to the copper and forms stabilizing interactions with the hydro­philic pocket, the CO_2_ binds in a ‘side-on’ conformation adjacent to the zinc and is stabilized by the hydro­phobic pocket (Fig. 2 ▸). The NO_2_^−^ is stabilized via hydrogen bonding to residues Thr199 and Thr200 while also interacting with W1 [Fig. 2 ▸(*b*)]. The CO_2_ binding shares the same hydrogen bond with Thr199 while also forming hydro­phobic interactions with Val121, Val143 and Trp209 [Fig. 2 ▸(*a*)]. The Cu–CAII active site retains the same W1, W2, W3a and W3b positions as Zn–CAII (Fig. 1 ▸). However, in Cu–CAII an extended ordered water network exists spanning past His64, forming a hydrogen-bonding network up to the second copper-binding site located at the N terminus. This network is achieved with the ordering of two additional waters compared with the Zn–CAII, named W4 and W5 [Fig. 1 ▸(*b*)]. Presumably, the additional water molecules complete a solvent network to span the two copper-binding sites, allowing the electron transfer necessary for the nitrite reductase reaction [Fig. 1 ▸(*b*)].

**Figure 3:**
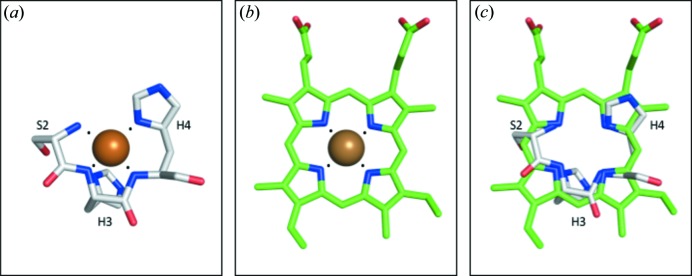
N terminus of Cu–CAII (T-1 site). (*a*) The T-1 copper is stabilized by the N terminus of Cu–CAII by residues Ser2, His3 and His4. The copper is also hydrogen bonded to solvent molecule facing His64 (presumably for electron transfer to the T-2 site). Interestingly, residue His3 adopts dual conformations, one away and one towards the copper (for electron density refer to Fig. S3). (*b*) Structure of an iron-containing porphyrin ring from *P. aeruginosa* nitrite reductase (PDB entry 1n15, Nurizzo *et al.*, 1999 ▸). (*c*) Superposition of Cu–CAII N terminus with the *P. aeruginosa* nitrite reductase heme with an RMSD of 0.27 Å. It is important to note that the N terminus T-1 site is less ordered in comparison to the rest of the structure. The occupancy and *B* factor of the T-1 site are 0.71 and 29.1 Å^2^ respectively, while for the T-2 site the occupancy and *B* factor are 1.00 and 11.4 Å^2^ respectively. This is because the N terminus needs to be transient, only forming when needed in the blood, effectively acting as an on/off switch. Also, this transient feature would allow rapid metal exchange, allowing trace metals in the blood to quickly bind and disassociate for electron transfer.

**Figure 4:**
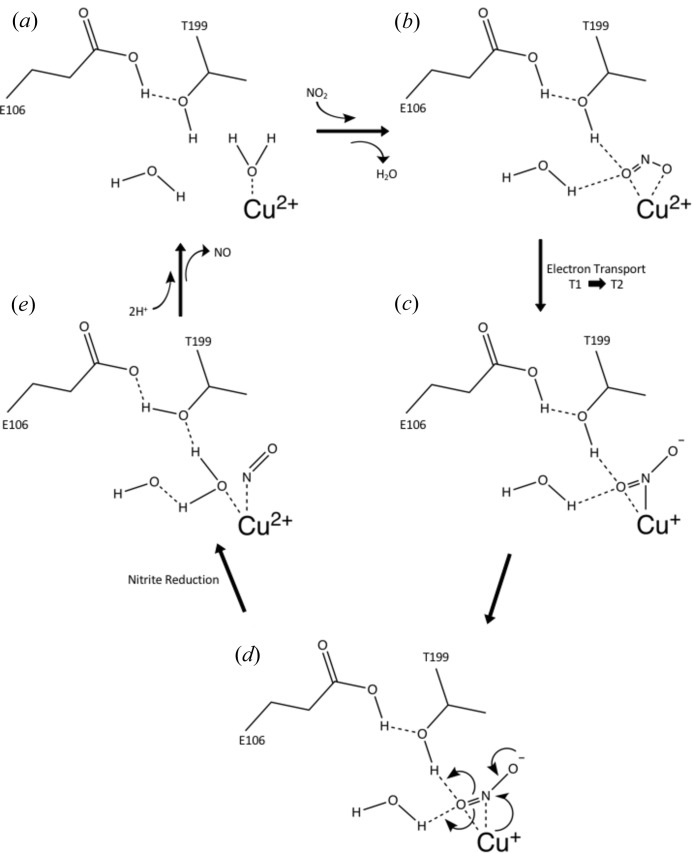
Proposed Cu–CAII nitrite reductase mechanism. (*a*) Cu–CAII in the resting state with a copper-bound solvent molecule. Thr199 is slightly acidic because of interactions with Glu106 allowing Thr199 to stabilize the solvent molecule. W1 is stabilized via hydrogen bonding to Thr200 and W2 (not shown for clarity). (*b*) NO_2_^−^ enters the active site, displacing the copper-bound solvent. NO_2_^−^ binds in a ‘hat’ conformation, with both oxygen atoms coordinating to the copper. One oxygen is primed for catalysis via hydrogen bonding to the hydroxyl of Thr199 and W1. (*c*) Intermolecular electron transfer from the T-1 copper site, donating an electron to the T-2 copper site and generating a Cu^+^ cation in the T-2 active site. This triggers a binding-mode change in NO_2_^−^ from ‘hat’ to ‘side-on’ coordination. One oxygen is uncoordinated from the copper and stabilized via the nitro­gen from Thr199 while the other oxygen retains hydrogen bonding to Thr199 and W1. (*d*) Reduction of nitrite begins via an electron donation from Cu^+^, resulting in a cascade of electron rearrangement and the regeneration of Cu^2+^. The primed oxygen accepts two protons from W1 and the acidic hydroxyl of Thr199 forming a bound water molecule. (*e*) The nitrite molecule is now reduced to nitric oxide and transiently bound to the Cu^2+^ cation along with the generated water molecule. As the water molecule forms, the nitric oxide is released from the copper. More protons are shuttled into the active site via the CA proton-shuttle His64 and the necessary catalytic protons are replenished regenerating the resting state in (*a*) (Duda *et al.*, 2001 ▸).

Mammalian CAIIs have a unique conserved N-terminus sequence (MSHHW) not observed in the other CA isoforms (https://www.uniprot.org/). However, as previously mentioned, this sequence is disordered in all the Zn–CAII crystal structures deposited in the Protein Data Bank [Fig. 3 ▸(*a*)] (Avvaru *et al.*, 2010 ▸; Saito *et al.*, 2004 ▸). The high-resolution structure of Zn–CAII (PDB entry 3ks3, Avvaru *et al.*, 2010 ▸) only shows order of the N terminus starting at His4, while the apo-CAII structures also show some order of His3 (Fig. S2). However, in the Cu–CAII structure, the N terminus becomes ordered, forming a pseudo-porphyrin ring, with the copper coordinated by several nitro­gens [Fig. 3 ▸(*a*)]. Previous work using paramagnetic nuclear magnetic resonance (NMR) techniques and X-ray absorption spectroscopy predicted this N-terminal structure as an amino terminal copper and nickel (ATCUN) binding motif as a high-affinity binding site for copper, *K*_d_ ≃ 0.5 n*M* (Nettles *et al.*, 2015 ▸). As confirmed from our X-ray crystallography, this structure acts as the T-1 copper site, coordinated to the main chain nitro­gens of Ser2, His3 and His4 [Fig. 3 ▸(*a*)]. It is more than likely that this site serves as the site of electron transfer to the T-2 copper active site [Fig. 1 ▸(*b*)]. The pseudo-porphyrin ring conformation, formed by the Cu–CAII N terminus has a striking resemblance to that of heme-containing nitrite reductases [Fig. 3 ▸(*b*)]. Structural superposition of the Cu–CAII N terminus with *Pseudomonas aeruginosa* nitrite reductase heme gave a root-mean-square deviation (RMSD) of 0.3 Å [Fig. 3 ▸(*c*)]. While not a porphyrin ring structure, this pseudo T-1 site, which has not previously been observed in metal–CAII structures, would provide the necessary electron donor site required for nitrite reduction. Hence, this structure provides an obvious mechanism for Cu–CAII to function as a nitrite reductase. Based on published mechanistic studies with bacterial nitrite reductase, the Cu–CAII active site has the T-1 and T-2 sites, the necessary bridging waters, and acidic residues to stabilize NO_2_^−^ and its intermediates, thus catalyzing nitrite reduction (Fig. 4 ▸) (Feng, 2012 ▸; Li *et al.*, 2015 ▸).

## 3. Discussion and conclusions   

As previously reported through NMR and X-ray crystallography studies, copper-substituted CAII has two binding sites for the metal cation, one in the canonical active site and one near the N terminus (Håkansson *et al.*, 1994 ▸; Nettles *et al.*, 2015 ▸). However, previous reports showed the secondary copper-binding site to be between His4 and His64, while our data, for the first time, shows a conformational change in the N terminus, forming a pseudo-porphyrin ring to bind a second copper. This new site is of mechanistic importance as the proton-shuttling residue His64 is free to undergo its conformational change required for carbonic anhydrase activity. Zn–CAII is known to be inhibited by copper, which coincides with the His4–His64 binding site reported by PDB entry 5eoi (Ferraroni *et al.*, unpublished work; Lionetto *et al.*, 2016 ▸). This work showed that if zinc is bound in the active site, copper binds through His64, thus proton transfer is inhibited. Interestingly, our work shows that if both sites are occupied by copper, His64 is unperturbed, allowing proton- or hypothetically electron-transfer between the two copper sites.

The water network within CAII has been extensively studied through structural and kinetic experiments (An *et al.*, 2002 ▸; Boone *et al.*, 2014 ▸; Duda *et al.*, 2003 ▸). Previous to this work, the ordered water network was thought to have stopped at the proton-shuttling residue His64. However, if copper is bound in both binding sites, we showed that the ordered water network further extends past His64, connecting the N-terminal binding site to the canonical binding site through a series of hydrogen bonds. Bacterial nitrite reductases are also known to have ordered waters within their active sites responsible for proton donation/acceptance and ordering substrate binding, similar to the water network of CAII (Li *et al.*, 2015 ▸). Marcus theory was originally developed to study the rate of electron transfer between ions (Marcus, 1964 ▸). The theory is used to determine activation energy in simple systems by calculating reorganization energies upon electron transfer. This takes into account the donor and acceptors’ size and distance apart, as well as dielectric constants and charge transferred (Marcus, 1964 ▸). This powerful tool can be used to study the dynamics and movement of electrons in solution. While originally only used for simple solutions, in the 1990s it was applied to large-scale biological systems, such as proteins, to calculate energy barriers in enzymatic function involved with electron transfer. However, in 1993 Dr Silverman applied Marcus theory to carbonic anhydrase to calculate the activation energy associated with proton transfer (Silverman, 2000 ▸). As previously mentioned, CA has an ordered water network with spanning hydrogen bonds that connect the zinc-bound hydroxyl to bulk solvent. With some modifications, Dr Silverman applied the Marcus theory to this water network to calculate the activation energy associated with the proton-transfer step of the CA mechanism (Silverman, 2000 ▸). He showed that this modified Marcus theory very accurately predicted the observed rates of CA proton transfer. However, Marcus theory has seen little success accurately predicting proton transfer except in one other enzyme, cytochrome *c* oxidase (Garbuz *et al.*, 2017 ▸). This protein, like CA, is involved with both proton and electron transfer. Perhaps the reason that Marcus theory so accurately predicts CA proton transfer is that the water network is optimized for both proton and electron transfer depending on the metal cation bound, thus making the theory interchangeably work with either electrons or protons.

While it is accepted that zinc-bound CAII exists in the blood, there is currently no direct experimental evidence to suggest the existence of copper-bound CAII. However, previous work from Aamand *et al.* showed that bovine CAII, when purified from bovine blood, has nitrite reductase activity (Aamand *et al.*, 2009 ▸). Furthermore, if this bovine CAII was dialyzed against EDTA, the nitrite reductase activity was ablated indicating that a metal cofactor within the bovine blood was needed for the CAII-dependent nitrite reductase activity (Andring *et al.*, 2018 ▸; Hanff *et al.*, 2018 ▸). The work presented here indicates that the metal cofactor is copper, thus strongly supporting the existence of Cu–CAII in blood. Furthermore, with the known concentration of copper in blood, paired with the high-affinity copper-binding sites, it is extremely likely that many CAII molecules exist with two bound copper atoms. Zinc is one of the most abundant trace metals in the blood, with typical concentrations of ∼96 µ*M*. However, copper also has relatively high concentrations in blood, typically ∼15 µ*M*, sevenfold lower than zinc (Schultze *et al.*, 2014 ▸). Previous work using a colorimetric 4-(2-pyridyl­azo)resorcinol assay showed that the CAII *K*_d_ for copper is 17 f*M*, while zinc *K*_d_ is 800 f*M*, indicating that CAII has 50-fold specificity toward copper over zinc (Hunt *et al.*, 1999 ▸). Furthermore, this research from Hunt *et al.* indicated that while the affinity is higher for copper than zinc, copper removal from the active site is facilitated through EDTA while EDTA has no effect on zinc removal from the active site (Hunt *et al.*, 1999 ▸). This coincides with the previous observation that EDTA prevented nitrite reductase activity (copper dependent) while not affecting carbonic anhydrase activity (zinc dependent) (Andring *et al.*, 2018 ▸). The high CAII affinity for copper over zinc is a well documented phenomenon with many papers proving that CAII’s affinity is much higher for copper than for zinc (Hunt *et al.*, 1999 ▸; Thompson *et al.*, 1999 ▸; McCall *et al.*, 2000 ▸, 2004 ▸), 
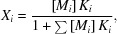
where *X*_*i*_ is the mole fraction of CA bound to the metal, *M_i_* is the molar concentration of total copper in blood and *K_i_* is the binding constant (*K*_a_) of Cu–CAII.

Using the calculations outlined by Thompson *et al.* and the affinities and concentrations from above, we can predict that ∼86% of CAII in the blood will have copper bound in the canonical active site (Thompson *et al.*, 1999 ▸). With the secondary site having an approximate *K*_d_ of 500 n*M* for copper, we suspect a substantial amount of bound Cu–CAII in the blood (Nettles *et al.*, 2015 ▸).

This study provides a feasible mechanistic view of how Cu–CAII can function as a nitrite reductase, given the physio­logical concentrations of CAII and copper in the blood. It would seem that CAII has the conformational ability to switch activity from a carbonic anhydrase to a nitrite reductase, dependent on the metal ion availability. Formation of Cu–CAII may explain nitrite reduction by cells under hypoxic conditions, allowing the formation of NO in RBCs. We feel these results clearly imply that we should re-examine the possible physiological role of Cu–CAII in blood.

## 4. Materials and methods   

Human CAII was expressed and purified according to previously published protocols (Pinard *et al.*, 2013 ▸; Tanhauser *et al.,* 1992 ▸). Briefly, a CAII gene-containing plasmid under the control of a T7 promoter was transformed into competent BL21 *Escherichia coli* cells via a standard BL21 transformation protocol. Following transformation, the *E. coli* cells were grown overnight in 100 ml of nutrient-rich Luria broth. Cells were then transferred to a large-scale 1 l culture in the presence of a selecting antibiotic and allowed to grow to an optical density of 0.6 at 600 nm. The cells were then induced by the addition of 0.5 m*M* iso­propyl β-d-1-thio­galactoside (IPTG) and 1 m*M* zinc sulfate and incubated for an additional 3 h. The zinc was added to aid in protein expression and folding, thus improving the yield. The cells were pelleted via centrifugation and subsequently lysed using a microfluidizer set to 18 000 PSI (1 PSI ≃ 6895 Pa). The Zn–CAII was purified from the cell lysate using affinity chromatography with a *p*-amino­methyl-benzene­sulfonamide affinity column. The final protein stock was buffer exchanged with storage buffer (50 m*M* Tris; pH 7.8) using a centrifugal filter. Purity was determined with SDS–PAGE and protein concentration was determined by UV–Vis spectroscopy at 280 nm.

In order to generate the copper-substituted CAII, the first step was to remove the zinc, generating apo-CAII. Immediately following purification, Zn–CAII was diluted to a concentration of 1 mg ml^−1^ in storage buffer and incubated with 5× chelation buffer (500 m*M* pyridine-2,6-di­carb­oxy­lic acid; 125 m*M* MOPS; pH 7.0). The solution was then gently stirred overnight at room temperature (20°C) and then passed over the *p*-amino­methyl-benzene­sulfonamide affinity column. Any residual Zn–CAII attached to the column, while the apo-CAII was collected in the flow through. The apo-CAII was buffer exchanged using centrifugal filters against storage buffer to remove any residual chelating agent. The loss of enzyme activity was verified using a standard colorimetric esterase based kinetic method, as described elsewhere (Uda *et al.*, 2015 ▸).

Apo-CAII crystals were grown via the hanging-drop vapor-diffusion method. Crystal trays were set up with 500 µl of mother liquor in the well, containing 1.6 *M* sodium citrate and 50 m*M* Tris at a pH of 7.8. Hanging drops of 9 µl were utilized consisting of a 1:1 ratio of 10 mg ml^−1^ protein to mother liquor. Crystal trays were left undisturbed at room temperature and apo-CAII crystal growth was observed after three days. To generate copper-substituted CAII crystals, the preformed apo-CAII crystals were incubated with 1 µl of a 10 m*M* stock solution of CuCl_2_ in the hanging drops. The addition of 10 m*M* CuCl_2_ did not result in osmotic shock to the crystals; however, concentrations greater than 50 m*M* resulted in cracked brittle crystals.

The Zn–CAII crystals were grown in the same fashion as the apo-CAII crystals. Crystal trays were set up with 500 µl of mother liquor in the well, containing 1.6 *M* sodium citrate and 50 m*M* Tris at a pH of 7.8. Then, 5 µl hanging drops were utilized consisting of a 1:1 ratio of 10 mg ml^−1^ protein to mother liquor. Crystal trays were left undisturbed at room temperature and Zn–CAII crystal growth was observed the next day.

The crystals were harvested utilizing MiTeGen loops, flash cooled in liquid nitro­gen and shipped to Stanford Synchrotron Radiation Lightsource (SSRL). Data were collected at the 9-2 beamline at SSRL using a PILATUS 6M detector with 0.3° oscillations, a wavelength of 0.9795 Å and a detector distance depending on the resolution of the crystal diffraction. Each dataset consisted of 600 images for a total of 180° of data. X-ray absorption spectroscopy was also performed at the 9-2 beamline to determine the presence of copper or zinc in the respective crystals (Figs. S4–S6).

The diffraction images were indexed and integrated using *XDS* then merged and scaled to the *P*2_1_ space group using the program *AIMLESS* via the *CCP*4 program suite (Kabsch, 2010 ▸; Evans & Murshudov, 2013 ▸). The diffraction data was phased with standard molecular replacement methods using the software package *Phenix* (Liebschner *et al.*, 2019 ▸) using the CAII PDB entry 3ks3 (Avvaru *et al.*, 2010 ▸) as the search model (Adams *et al.*, 2010 ▸). Coordinate refinements were calculated using *Phenix*, while the program *Coot* was utilized to add solvent molecules and make individual real-space refinements of each residue when needed (Adams *et al.*, 2010 ▸; Emsley & Cowtan, 2004 ▸). Figures were generated in the molecular graphical software *PyMOL* and protein–ligand interactions and bond lengths were determined using *LigPlot*+ (Schrödinger, LLC; Laskowski & Swindells, 2011 ▸). For the crystallographic data-collection and refinement statistics refer to Table S1 in the supporting information. The apo- and Cu–CAII structures have been deposited in the PDB with accession numbers 6pea and 6pdv, respectively.

## Supplementary Material

Supporting table and figures. DOI: 10.1107/S2052252520000986/lz5032sup1.pdf

PDB reference: copper–carbonic anhydrase II, a nitrite reductase, 6pdv

PDB reference: high-resolution apo-carbonic anhydrase II, 6pea
