# Structure and mechanism of copper–carbonic anhydrase II: a nitrite reductase. Corrigendum

**DOI:** 10.1107/S2052252521000208

**Published:** 2021-01-18

**Authors:** Jacob T. Andring, Chae Un Kim, Robert McKenna

**Affiliations:** a Department of Biochemistry and Molecular Biology, College of Medicine, University of Florida, Gainesville, FL 32610 USA; b Department of Physics, Ulsan National Institute of Science and Technology (UNIST), Ulsan 44919, Republic of Korea

**Keywords:** catalytic metal ions, copper–carbonic anhydrase II, apo-carbonic anhydrase II, nitrite reductases, nitric oxide, X-ray crystallography

## Abstract

Statements in the article by Andring *et al.* [*IUCrJ*, (2020), **7**, 287–293] are corrected.

## Abstract 2

Corrigendum to the article by Andring *et al.* [*IUCrJ*, (2020), **7**, 287–293].

The following statements in the article by Andring *et al.* (2020 ▸) are corrected:

(*a*) On page 288, ‘recent reports have shown that CAII can also reduce nitrite (NO_2_^−^ **)** to nitric oxide (NO), and thus, may play a role in vasodilation and regulation of blood pressure (Andring *et al.*, 2018 ▸; Aamand *et al.*, 2009 ▸; Hanff *et al.*, 2018 ▸).’ should read ‘recent reports have shown that CAII can also reduce nitrite (NO_2_^−^ **)** to nitric oxide (NO), and thus, may play a role in vasodilation and regulation of blood pressure (Aamand *et al.*, 2009 ▸).’. Hanff *et al.* (2018 ▸) did not show reduction to NO but instead showed nitrite dehydratase activity, which is not a redox reaction.

(*b*) On pages 288–299, ‘However, when dialyzed with ethylenediaminetetraacetic acid (EDTA), the enzyme retained its carbonic anhydrase activity yet lost its nitrite reductase activity (Hanff *et al.*, 2018 ▸)’ should read ‘However, when dialyzed with ethylenediaminetetraacetic acid (EDTA), the enzyme retained its carbonic anhydrase activity yet lost its nitrite reductase activity (Andring *et al.*, 2018 ▸)’.

(*c*) And on page 291, ‘... indicating that a metal cofactor within the bovine blood was needed for the CAII-dependent nitrite reductase activity (Andring *et al.*, 2018 ▸; Hanff *et al.*, 2018 ▸)’ again should not cite Hanff *et al.* (2018 ▸) and should read ‘... indicating that a metal cofactor within the bovine blood was needed for the CAII-dependent nitrite reductase activity (Andring *et al.*, 2018 ▸)’.
