# Supplementary material for: Structure and mechanism of copper–carbonic anhydrase II: a nitrite reductase
Source: IUCrJ. 2020 Feb 21;7(Pt 2):287–93. doi: 10.1107/S2052252520000986 (PMC7055381; doi:10.1107/S2052252520000986)
Supplement: Supplementary file 1 [file m-07-00287-sup1.pdf]

IUCrJ

Volume 7 (2020)

Supporting information for article:

Structure and mechanism of copper–carbonic anhydrase II: a nitrite reductase

Jacob T. Andring, Chae Un Kim and Robert McKenna

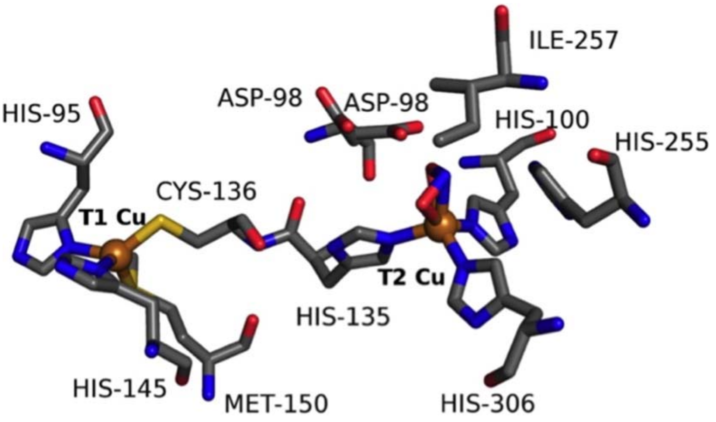

Figure S1 T1 and T2 Copper Binding Sites in *Achromobacter cycloclastes* Cu Nitrite Reductase. T1 and T2 copper sites with endogenously bound NO_2^- in T2 site. Adapted from Li *et al.* (2015) with permissions. Copyright 2015 American Chemical Society.

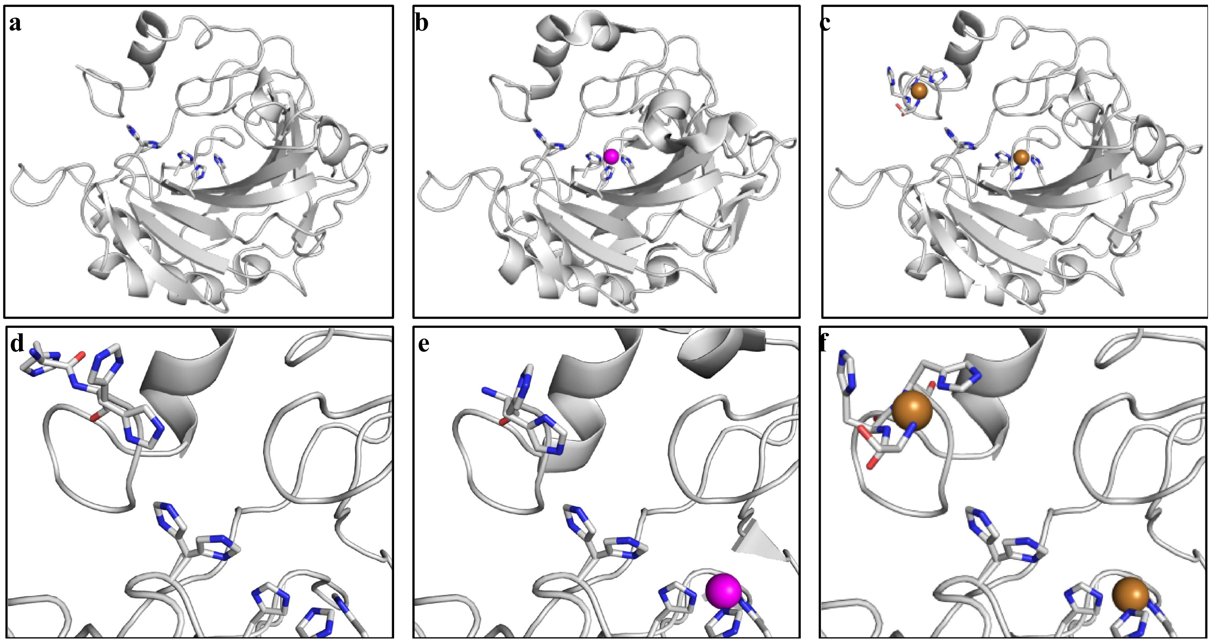

Figure S2 Metal binding in CA II. **A** and **D** Apo-CA II after treatment with DCA. The active site is empty and the N-terminus is disordered. **B** and **E** the Zn-CA II active site with zinc chelated by H94, H96, and H119. The N-terminus is disordered with density only for H4. **C** and **F** Cu-CA II with metal bound at both the T1 and T2 sites. The N-terminus is ordered around the copper atom, forming a ATCUN binding site.

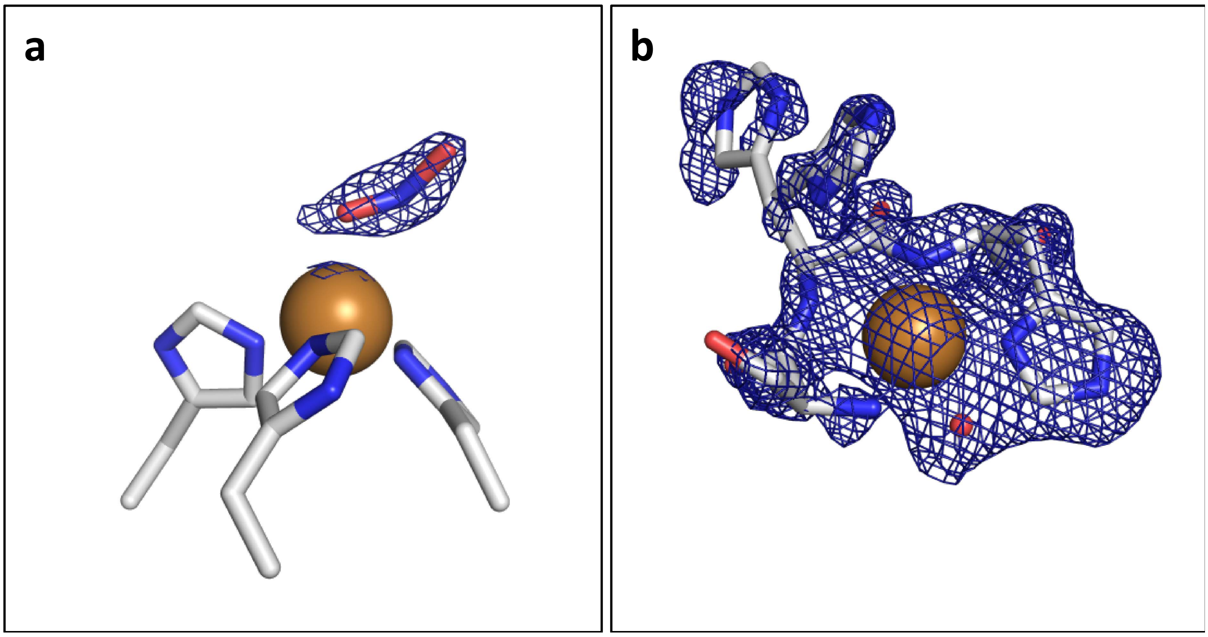

Figure S3 Electron density for Cu-CA II T1 and T2 sites. **A** Electron density for the NO_2^- bound at T2 site in copper substituted CA II, contoured to 1.5 sigma. **B** Electron density for the T2 site in Cu-CA II contoured to 0.8 sigma.

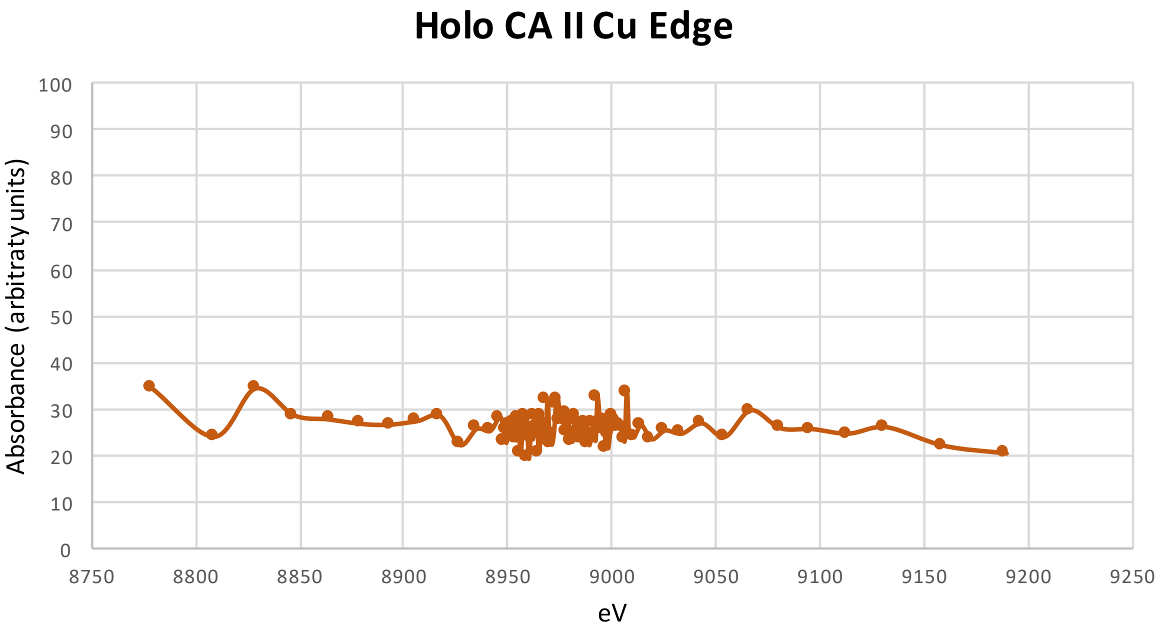

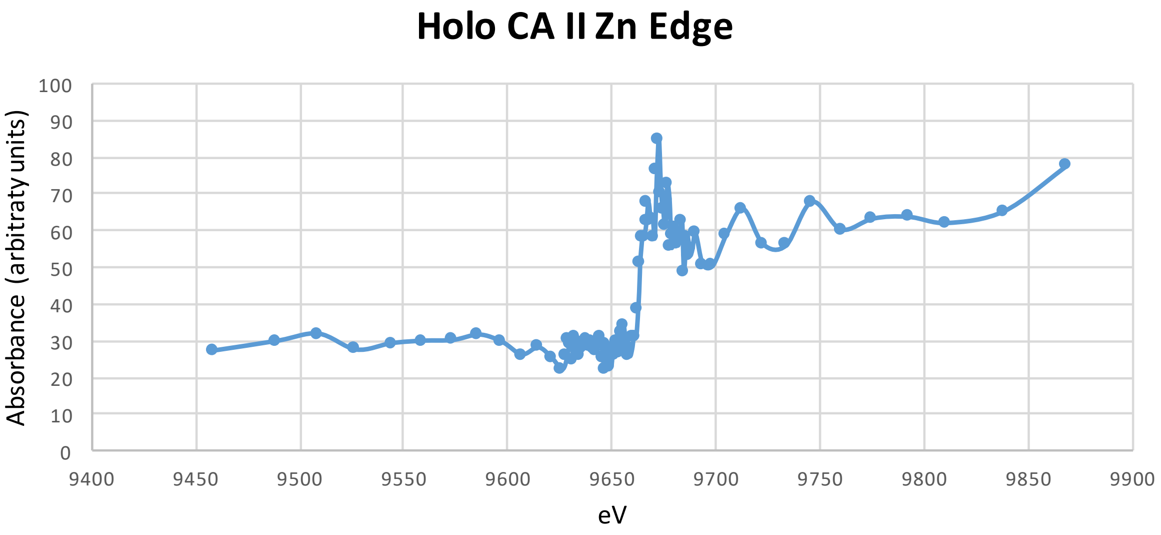

Figure S4 X-ray absorption edge spectra of Zn-CA II. Zn-CA II shows the expected absorption edge at ~9659 eV indicative of zinc bound.

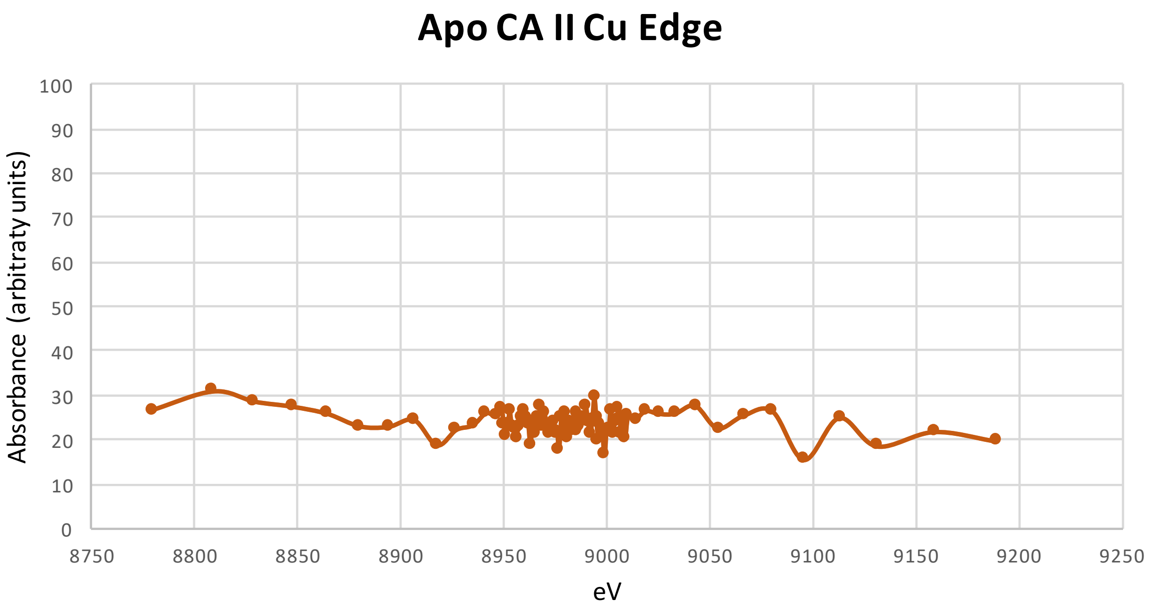

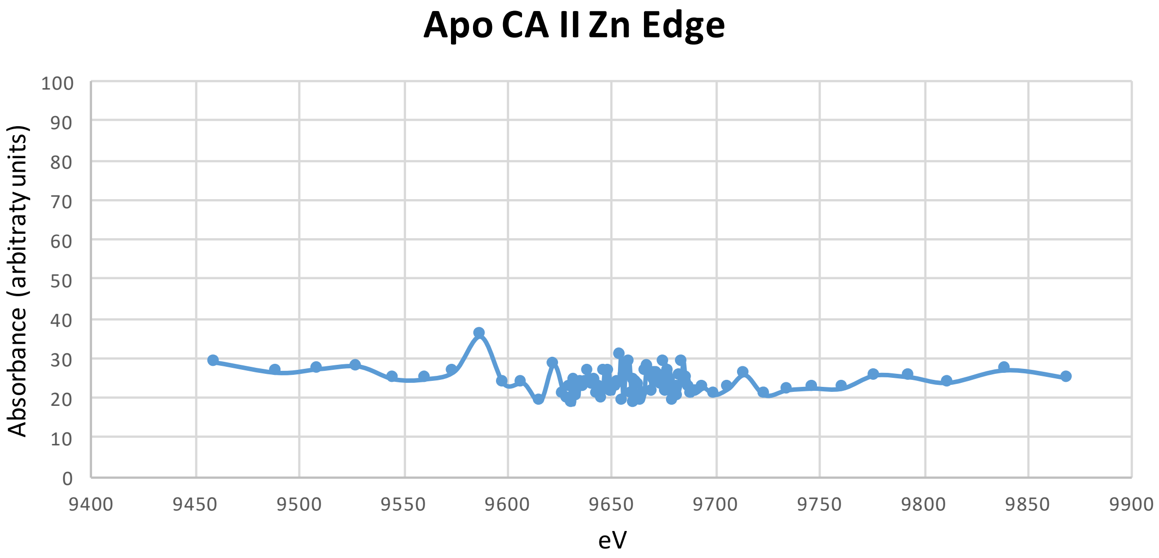

Figure S5 X-ray absorption edge spectra of Apo-CA II. Apo-CA II shows no absorption edge around 8979 eV nor 9659 eV indicating no metal present.

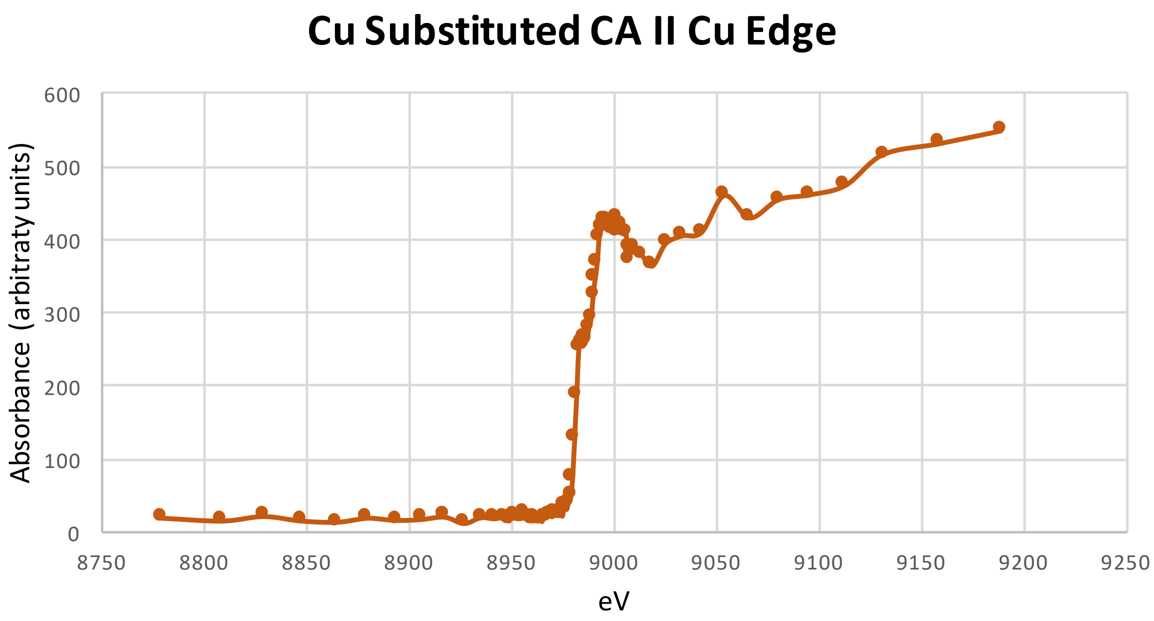

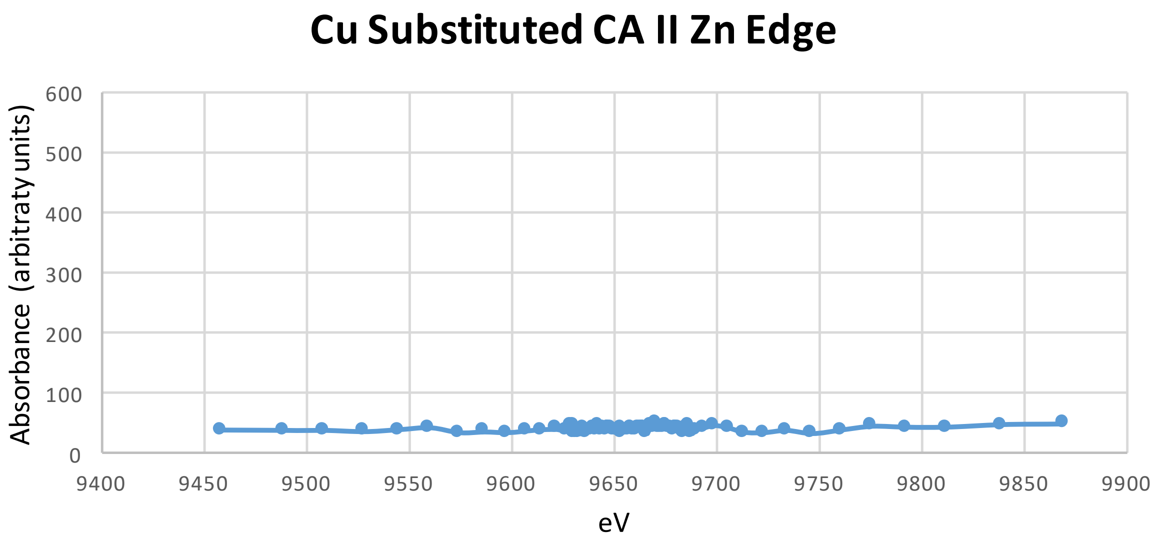

Figure S6 X-ray absorption edge spectra of Cu-CA II. Cu-CA II shows the copper absorption edge at ~8979 eV but not the zinc edge at 9659 eV indicating only copper bound.

Table S1 Data collection and refinement statistics.

Values in parentheses correspond to those of the highest-resolution shell.

	**Apo CAII**	**Cu CAII**
**Wavelength (Å)**	0.9795	0.9795
**Resolution range (Å)**	39.88 - 1.36 (1.41 - 1.36)	34.90 - 1.23 (1.27 - 1.23)
**Space group**	P 1 21 1	P 1 21 1
**Unit cell: a,b,c (Å)**<br>**α, β, ɣ (°)**	41.3, 42.3, 72.0<br>90, 104.2, 90	41.2, 42.4, 72.0<br>90, 104.3, 90
**Total reflections**	163082 (11244)	379527 (20966)
**Unique reflections**	49501 (4044)	68889 (6488)
**Multiplicity**	3.3 (2.8)	5.5 (3.2)
**Completeness (%)**	95.9 (76.5)	98.1 (92.8)
I/I_{σ}	30.6 (6.3)	12.9 (1.6)
**Wilson B-factor (Å^2)**	11.9	13.1
R_{merge}[a] **(%)**	2.17 (17.24)	8.98 (63.41)
R_{work}[b] **(%)**	14.86 (18.83)	15.68 (27.71)
R_{free}[c] **(%)**	16.41 (20.91)	17.41 (28.60)
R_{pim}[d] **(%)**	1.40 (12.18)	3.92 (41.76)
**Reflections used in refinement**	50381 (4045)	68841 (6471)
**Reflections used for R-free**	2005 (156)	1846 (180)
**Number of non-hydrogen atoms**	2375	2406
**macromolecules**	2156	2188

**ligands**	8	13
**solvent**	211	205
**Protein residues**	258	262
**RMS(bonds) (Å)**	0.007	0.014
**RMS(angles) (°)**	1.29	1.74
**Ramachandran favored (%)**	97.3	95.7
**Ramachandran allowed (%)**	2.7	4.3
**Ramachandran outliers (%)**	0	0
**Rotamer outliers (%)**	0.4	0
Average B-factor($Å^2$)	17.2	19.2
macromolecules	16.2	18.4
ligands	35.8	29.1
solvent	26.7	27.6

[a]$R_{merge} = (\Sigma|I - \langle I\rangle|/\Sigma \langle I\rangle) \times 100$.

[b]$R_{\mathrm{work}} = (\Sigma|F_{\mathrm{o}} - F_{\mathrm{c}}|/\Sigma|F_{\mathrm{o}}|) \times 100$.

[c]R_{free} is calculated in the same way as R_{cryst} except it is for data omitted from refinement (5% of reflections for all data sets).

[d]$R_{\mathrm{pim}} = [(\Sigma\surd 1/N - 1)\Sigma|I - \langle I\rangle|/\Sigma \langle I\rangle] \times 100$.
